# Transfer in Motor Sequence Learning: Effects of Practice Schedule and Sequence Context

**DOI:** 10.3389/fnhum.2015.00642

**Published:** 2015-11-24

**Authors:** Diana M. Müssgens, Fredrik Ullén

**Affiliations:** Department of Neuroscience, Karolinska InstitutetStockholm, Sweden

**Keywords:** skill learning, movement sequence, serial reaction time, practice schedule, skill transfer, contextual interference

## Abstract

Transfer (i.e., the application of a learned skill in a novel context) is an important and desirable outcome of motor skill learning. While much research has been devoted to understanding transfer of explicit skills the mechanisms of skill transfer after incidental learning remain poorly understood. The aim of this study was to (1) examine the effect of practice schedule on transfer and (2) investigate whether sequence-specific knowledge can transfer to an unfamiliar sequence context. We trained two groups of participants on an implicit serial response time task under a Constant (one sequence for 10 blocks) or Variable (alternating between two sequences for a total of 10 blocks) practice schedule. We evaluated response times for three types of transfer: task-general transfer to a structurally non-overlapping sequence, inter-manual transfer to a perceptually identical sequence, and sequence-specific transfer to a partially overlapping (three shared triplets) sequence. Results showed partial skill transfer to all three sequences and an advantage of Variable practice only for task-general transfer. Further, we found expression of sequence-specific knowledge for familiar sub-sequences in the overlapping sequence. These findings suggest that (1) constant practice may create interference for task-general transfer and (2) sequence-specific knowledge can transfer to a new sequential context.

## Introduction

Learning new motor skills can take a considerable amount of time and effort. Therefore, it is often desirable that a newly learned skill can also be applied outside the specific context within which it was acquired. Motor skill transfer (i.e., the application of a learned skill in a new task or context) is thus an important aspect of motor learning. Transfer can be described along several dimensions, such as positive vs. negative or broad vs. narrow (Adams, 1987; Schmidt and Lee, 2005). Positive transfer is seen when training of one skill facilitates performance in another, novel situation. Negative transfer is the opposite phenomenon, where earlier training interferes with performance on a new task. In narrow transfer, such influences are seen between similar tasks, while in broad transfer, training effects are seen on a wide range of tasks. Finally, certain skills may, or may not, transfer between effectors (i.e., they may be more or less specific to the effector with which they were trained). In most training scenarios one would thus want to achieve broad positive skill transfer, potentially also between effectors.

One factor that influences the amount of skill transfer is the training schedule. Variable training schedules (e.g., randomizing or alternating between different tasks) typically lead to greater performance retention and transfer than blocked schedules (Shea and Morgan, 1979; Magill and Hall, 1990). This phenomenon, termed contextual interference (CI), has been observed for a variety of motor tasks such as explicit visuo-motor sequence learning (e.g., Shea and Morgan, 1979; Wymbs and Grafton, 2009; Tanaka et al., 2010), handwriting (Ste-Marie et al., 2004), simple drawing tasks (Albaret and Thon, 1998), various sports skills (Wrisberg and Liu, 1991; Hall et al., 1994; Douvis, 2005, but see: Brady, 2008), and certain other complex tasks such as bimanual coordination (Pauwels et al., 2014) and rotatory pursuit skills (Heitman et al., 2005). One prominent theory on the mechanisms of CI argues that variable practice is advantageous because each switch between tasks requires the effortful reconstruction of motor plans in working memory (Lee and Magill, 1983; Immink and Wright, 1998; Cross et al., 2007). This repeated planning and updating of movement parameters is more attention demanding (Li and Wright, 2000), which is thought to eventually lead to more persistent skill representations in long-term memory. However, this explanation does not account for more recent findings from studies demonstrating superior skill retention after variable practice also for more implicit tasks such as incidental motor sequence learning (Song et al., 2012, 2015; Lin et al., 2013).

Transfer in implicit motor learning seems to be rather narrow and inflexible, with no transfer being observed, e.g., after changes in response locations (Willingham et al., 2000) or stimulus–response associations (Schwarb and Schumacher, 2010). Even changes in task-irrelevant aspects of the visual context in which a motor sequence is presented can be detrimental for implicit skill transfer (Jiménez et al., 2006; Abrahamse and Verwey, 2008). Jiménez et al. (2006) showed that changing superficial task parameters by adding task-irrelevant distractor stimuli abolished implicit skill transfer. Yet, despite of being rather inflexible with regards to superficial changes in stimulus presentation, implicit skills seem to be more robust than explicit skills if the sequential context of a task is abruptly changed. When Jiménez et al. (2006) trained participants on a sequential motor task and then changed the stimulus presentation to a random order (experiments 3 and 4), they observed an expected worsening of participants’ performance. However, at certain points the random sequence was interspersed with the previously trained sequence. Participants who had learned the sequence implicitly, but not those who had learned it explicitly, showed evidence of sequence transfer as their performance recovered on those sections that contained the familiar sequence. This suggests that expression of implicit sequence knowledge might be triggered by the immediately preceding (familiar) sequence context, even if the familiar sequence itself is embedded within an unfamiliar (random) sequence context. However, given that the interspersed familiar segments in that study consisted of the entire training sequence it is not clear whether the context of the entire trained sequence is necessary or if sequence-specific knowledge can also transfer to familiar sub-sequences that are embedded within an unfamiliar sequence context.

Based on the previously mentioned findings of a CI effect on implicit motor sequence learning (Song et al., 2012, 2015; Lin et al., 2013) one might expect a similar benefit of variable practice for motor sequence transfer. To understand how different training schedules could affect skill transfer it is important to distinguish between different types of transfer. Generally, transfer in motor sequence learning tasks can be divided into sequence-specific and sequence non-specific components. Transfer is considered to be sequence-specific if performance improvements on a transfer task can be attributed to knowledge of the sequential order of the task elements. Sequence-specific knowledge can, depending on the exact task parameters, be represented in various formats such as stimulus-based coordinates (e.g., Remillard, 2003; Clegg, 2005), effector-based coordinates (e.g., Kami et al., 1995; Bischoff-Grethe et al., 2004; Park and Shea, 2005; Verwey and Clegg, 2005), response location based coordinates (e.g., Willingham et al., 2000; Witt and Willingham, 2006), or in terms of response effects (e.g., Ziessler and Nattkemper, 2001; Stocker et al., 2003; Stocker and Hoffmann, 2004) or the relationship between consecutive responses (e.g., Koch and Hoffmann, 2000; Hoffmann et al., 2001). Transfer of sequence non-specific (task-general) skills refers to performance improvements in task components that are not dependent on knowledge of the sequential structure, such as improvements in visual stimulus processing, stimulus–response mapping, or motor command generation. To distinguish between sequence-specific and task-general contributions to skill transfer it is thus necessary to compare transfer in tasks that contain familiar sequence information with transfer in similar tasks that do not contain familiar sequence structures.

Another dimension of skill transfer is effector specificity (e.g., whether a sequential skill transfers between hands). Although inter-manual skill transfer can again be divided into sequence-specific and non-specific transfer a number of studies have shown rather large inter-manual transfer effects for sequential knowledge (Willingham et al., 2000; Grafton et al., 2002; Verwey and Clegg, 2005; Berner and Hoffman, 2009). Inter-manual transfer is likely to benefit from both increased sequence-specific transfer and from improvements in certain task-general aspects such as stimulus processing or the mapping between stimuli and their relative response locations. Thus, if CI affects any of these components one would expect to see an advantage of variable practice also for inter-manual transfer. Yet, the effects of practice schedule onto implicit inter-manual transfer have – to our knowledge – not been studied so far.

One task that is commonly used to investigate sequence learning and transfer is the serial response time (SRT) task (Nissen and Bullemer, 1987). In an SRT task stimuli appear at different spatial locations and participants respond by pressing a button corresponding to the location of the stimulus. Unbeknownst to the participants, the stimuli follow a sequential order during training and performance improvements are quantified as reductions in response time (RT). After several training blocks a random sequence is introduced and the RT difference between the last training (sequential) block and the random block is typically attributed to sequence-specific learning. RT decreases in the random block, relative to the first block, are considered sequence non-specific improvements.

When quantifying transfer it is important to carefully choose the training and transfer sequences to avoid confounds due to differences in sequence difficulty or saliency. A methodological challenge is that RT differences between different sequences could reflect learning of both complex sequential structures and simpler statistical regularities (e.g., frequencies of elements and transitions) of the training sequence. In one influential study, Reed and Johnson (1994) used second-order conditional sequences (i.e., sequences where the identity of a given element is determined by the two preceding elements, but not by one element alone) to specifically test whether complex sequence structures can be learned implicitly. Importantly, training and control sequences were matched on a variety of properties, so that knowledge of sequence structure could be disentangled from knowledge of statistical regularities.

Here, we used a similar approach as Reed and Johnson (1994) by employing sequences that were carefully matched in terms of salient structural properties (see Materials and Methods) to investigate different types of transfer effects after constant and variable training. Participants were divided into two groups which received either constant training of a single sequence, or variable (alternating) training of two sequences. Transfer effects were evaluated by comparing performance on three different test sequences before and after training. One test sequence (T0) had no structural overlap with either of the training sequences and thus served to quantify sequence non-specific transfer effects. A second sequence (T3) had partial structural overlap (three shared triplets) with each of the training sequences. This sequence was used to investigate sequence-specific transfer for familiar sub-sequences embedded into an unfamiliar sequence context. A third sequence (TrL) was perceptually identical to the trained sequence but was performed with the opposite (untrained) hand. This sequence was used to investigate inter-manual transfer in extrinsic coordinates. Contrary to transfer sequence T3, where familiar sub-sequences were embedded into novel sequence context, the sequence context for transfer sequence TrL was thus entirely familiar.

We investigated two hypotheses. First, we tested whether variable sequence training leads to greater skill transfer than constant training. As outlined above, variable practice has been found to be advantageous for a variety of motor learning tasks including relatively simple explicit tasks, more complex sports and real-life tasks, and implicit sequence learning tasks. We thus predicted that the Variable practice group would show larger performance improvements on the non-overlapping sequence (T0, sequence-unspecific transfer), as well as on the trained sequence performed with the left hand (TrL, inter-manual transfer) and on the structurally overlapping sequence (T3, sequence-specific transfer). Secondly, we hypothesized that structure-specific knowledge partly transfers to new sequences that contain fragments of the trained sequence. Specifically, we expected that (i) transfer effects would be larger for the partially overlapping sequence (T3) than for the non-overlapping sequence (T0) and (ii) that within the partially overlapping sequence transfer would be specifically larger for predictable elements than for corresponding unpredictable elements.

## Materials and Methods

### Ethical Statement

All participants gave written, informed consent to participate and the study was approved by the Regional Ethical Review Board in Stockholm, Sweden (Dnr. 2012/198-32/4).

### Participants

Participants were recruited through posters displayed at the Karolinska Institutet campus and through the website Studentkaninen (www.studentkaninen.se), a Swedish website for research volunteers. Sixty individuals initially participated in the study. Due to technical problems, we did not obtain data from the left hand task in two participants. These participants were excluded from all analyses involving the left hand task, but their data was included in all other analyses. Further, one individual was excluded from the analyses, because of exceptionally slow RTs (2.5–3.8 SD above the sample mean in all tasks). Three additional participants were excluded, because they showed no learning of the experimental sequence Tr1 in the Training session (i.e., the linear regression of RT on trial number had a positive slope). The final analyses thus included 56 participants in all tasks involving the right hand and 54 participants in all left-hand tasks. The age of these participants ranged from 19 to 43 years (mean = 27.8, *SD* = 5.5); 28 participants were male. All participants were right-handed and reported to be free of any neurological or psychiatric conditions.

**FIGURE 1:**
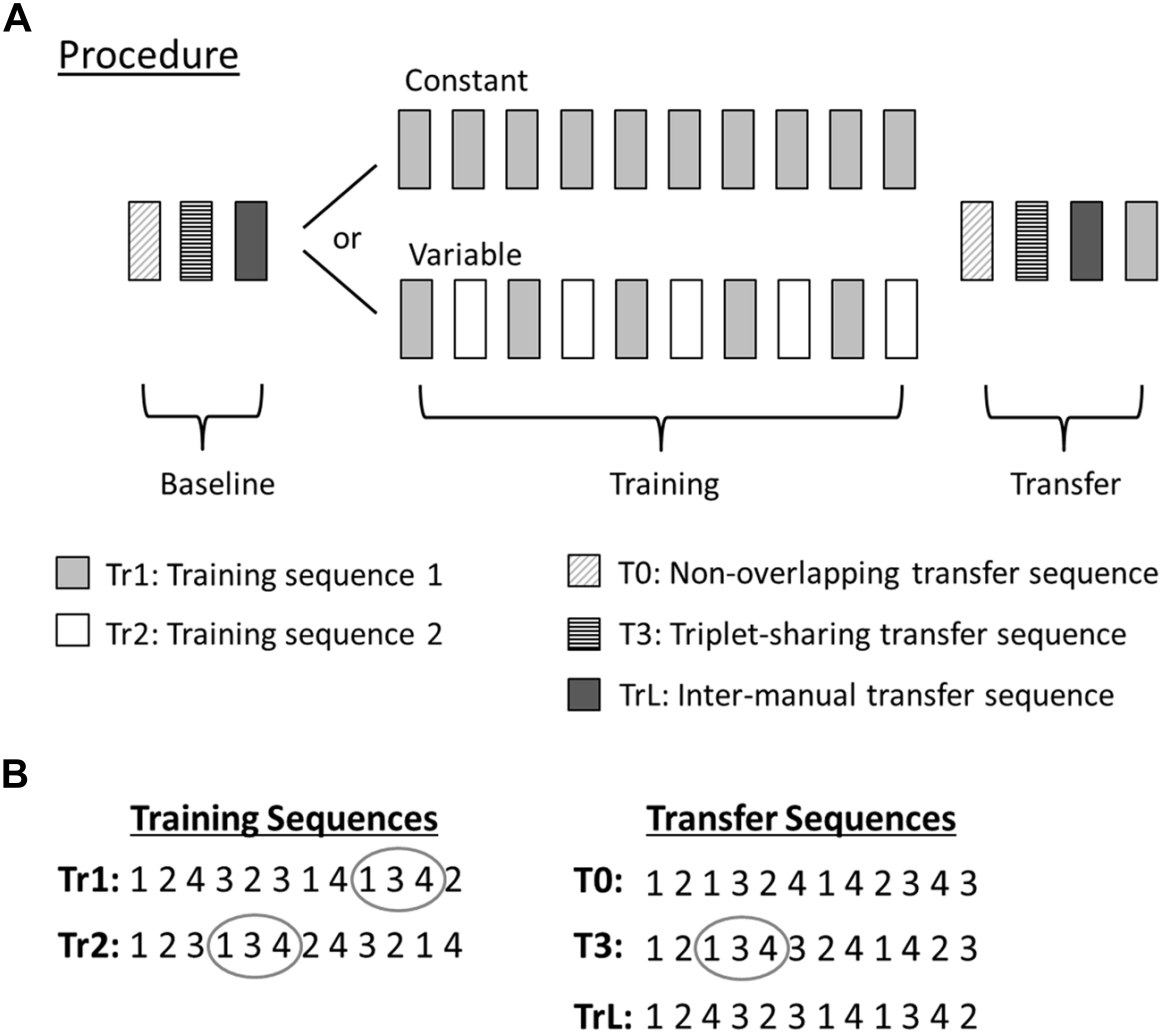
**Experimental procedure and sequential stimuli. (A)** The experimental procedure consisted of three sessions: Baseline, Training, and Transfer. The Baseline session consisted of one block of each of the three transfer sequences (T0, T3, TrL) and the Transfer session consisted of the same three sequences (presented in the same order as during Baseline) plus one additional block of Tr1 at the end of the session. Block order during Baseline was randomized across participants and counterbalanced between groups. In the Training session the Constant group performed 10 blocks of the Tr1 sequence and the Variable group alternated between Tr1 and Tr2 blocks. All blocks were separated by 20 s of rest, both within and between sessions. Each block contained 10 uninterrupted sequence repetitions, thus requiring a total of 120 responses (12 sequence elements × 10 repetitions) per block. **(B)** Sequence structure of the training (left) and transfer (right) sequences. The training sequences (Tr1 and Tr2) shared six triplets with each other, three of which were also shared with sequence T3 (one example of a shared triplet encircled). None of the training sequences shared any triplets with sequence T0.

Participants were randomly assigned to either the Constant group (*n* = 28, mean age = 27.9), which practiced a single sequence during the training session, or to the Variable group (*n* = 28, mean age = 27.7), which practiced two different sequences alternatingly (**Figure 1A**).

### Sequential Tasks

Stimulus presentation and data collection were performed on a PC, using a script written in the E-Prime software package (Psychological Software Tools, Inc.). Stimuli were presented on the computer monitor and responses were collected from the computer keyboard. Participants stayed seated in front of the computer during the whole experiment and were allowed to adjust the position of the keyboard and chair. The identity and timing of all stimuli and responses were saved to a log file.

The experiment consisted of a number of SRT tasks (Nissen and Bullemer, 1987; i.e., series of four-choice RT trials). Four empty squares – corresponding to sequence elements 1, 2, 3, and 4 – were presented in a horizontal arrangement along the middle of the computer monitor. On each trial, one of the squares turned yellow and remained yellow until the participant pressed the correct key. Four different response keys (H, U, I, L for the index to little finger of the right hand and G, R, E, A for the index to little finger of the left hand) were used, corresponding to the four stimulus locations. As soon as the participant gave the correct response the next stimulus appeared; thus, the response-to-stimulus interval was 0 ms. We chose this interval because the absence of a response-to-stimulus delay has been previously shown to reduce explicit sequence awareness (Destrebecqz and Cleeremans, 2001). If no correct response was registered within 2 s the program continued automatically with the next stimulus. The experiment was described as a “reaction time task” and participants were instructed to respond to the stimuli as quickly and as accurately as possible. Participants were not told that the stimuli would appear in a sequential order.

Stimuli always followed a repeating, deterministic sequence of 12 elements. The tasks were administered in blocks consisting of 10 uninterrupted repetitions of the same sequence, (i.e., 120 RT trials per block). Four different sequences were used in different tasks: Tr1, Tr2, T0, and T3 (**Figure 1B**). Sequence Tr1 was also used in a left-hand task, TrL. When comparing performance or transfer between different sequences, it is essential that the sequences are matched on various properties that are likely to influence learning (Reed and Johnson, 1994). The sequence structure of the employed sequences is shown in **Figure 1B**. All sequences were second-order conditional sequences (i.e., each element is uniquely predicted by the preceding bigram of two consecutive elements, but never by one preceding element alone). There were no immediate repetitions of elements. The frequencies of all individual elements (1, 2, 3, 4) were the same (0.25) in all sequences, as were the frequencies (0.083) of all of the 12 allowed bigrams (12, 13, 14, 21, 23, 24, 31, 32, 34, 41, 42, 43). The sequences were also matched on other putatively salient properties that could influence performance (Reed and Johnson, 1994): reversal frequency (0.25; i.e., the frequency of palindromic triplets with a ‘back-and-forth’ structure, such as in 121), rate of full coverage (5.08; i.e., the average number of elements encountered before each element has occurred at least once), and rate of full transition usage (13; i.e., the average number of elements encountered before each possible transition has occurred once).

Furthermore, the sequences were constructed such that comparisons between the different tasks would be informative about the nature of possible transfer effects. Sequences Tr1 and Tr2 were used as training sequences. The Constant group trained only Tr1 and the Variable group trained alternatingly on Tr1 and Tr2. Sequence T0 shared no triplets with Tr1 or Tr2. Performance on this sequence could thus provide information about sequence non-specific transfer effects. We chose a deterministic, rather than random control sequence, so that we could exclude the possibility of any accidental structural overlap between T0 and the training sequence. Moreover, this enabled us to control the sequence for all of the above mentioned statistical sequence regularities. Sequence T3 shared the same three triplets (134, 231, and 432) with both Tr1 and Tr2 and was used to investigate sequence-specific transfer effects. Since the shared triplets appeared at different ordinal positions in T3 than in Tr1 and Tr2 sequence-specific transfer should only be observed if sequence knowledge for the smallest unique sub-parts (i.e., triplets) is still preserved if triplets are isolated from their sequence context and embedded into an unfamiliar sequence context.

Finally, to investigate inter-manual transfer, a task TrL was included, where participants performed the Tr1 sequence using their left hand. Inter-manual transfer was evaluated in extrinsic coordinates, meaning that both the order of visual stimulus locations and the mapping between visual stimuli and their relative response locations (i.e., leftmost stimulus to leftmost response location, rightmost stimulus to rightmost response location) were the same as for the training sequence.

### Experimental Procedure

All experiments were performed in a quiet room. Before the start of the experiment each participant made 12 practice responses with each hand to become familiar with the task. The order of these responses was not related to any of the sequences. The experiment consisted of three sessions: baseline, training, and transfer (**Figure 1A**). The baseline session included one block of each of the TrL, T0, and T3 tasks. The order of these three tasks within the baseline session was randomized across participants and counterbalanced between groups to prevent any possible task-order effects.

Participants then performed the training session, which was organized differently for the two groups. The Constant training group performed 10 blocks of the Tr1 task. The Variable group also performed a total of 10 blocks, but alternated between blocks of Tr1 and Tr2, thus yielding a total of five blocks per task.

The final transfer session included one block each of the TrL, T0, T3, and Tr1 tasks. Task order within the transfer session was the same as during baseline, except for the additional block of Tr1 which was always presented at the end of the session. Participants were not informed that the experiment consisted of three sessions. All blocks were separated by 20 s of rest, both within and between sessions to avoid any noticeable distinction between sessions.

### Questions on Explicit Sequence Knowledge

After completion of the three sessions participants filled out a paper-and-pencil questionnaire related to their sequence awareness. First, they were asked a two-choice question whether they had perceived any pattern in the presented stimuli: “Did you notice any regularity in the presentation of the yellow squares?”, with response alternatives “Yes” and “No”. In the second question they were asked to rate how sure they were that there was a pattern or sequence in the stimuli: “On a scale from 1 (not sure at all) to 10 (very sure) can you indicate how sure you were that there was a pattern or sequence in the presentation of the yellow squares?”

### Statistical Analyses

**FIGURE 2:**
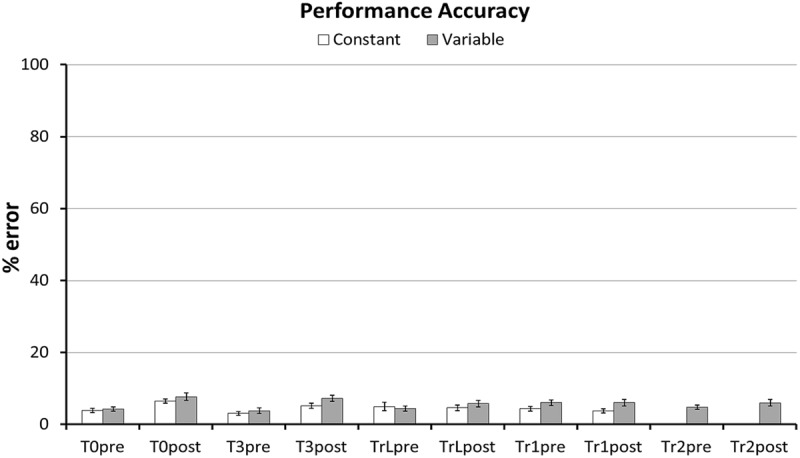
**Performance errors per task.** Average percent of incorrect responses per task and Group. Error bars represent standard error of the mean. Incorrect responses varied between 3.0 and 7.7% (3.6–9.2 errors out of 120 responses) per block and were removed before further analysis.

Data were pre-processed using custom-written scripts in MATLAB (version R2013b, The MathWorks, Inc., Natick, MA, USA) and analyzed in SPSS (version 21.0 for Windows, IBM Corp., Armonk, NY, USA). For each participant we excluded wrong responses and calculated the median RT per block. The average percentage of excluded (i.e., wrong) responses varied between 3.0 and 7.7% per block (**Figure 2**). For each sequence we calculated an Improvement Score, defined as RT at baseline – RT at transfer, to quantify RT changes across sessions. Since RTs were approximately normally distributed in each group, except for Tr1 Improvement Scores in the Constant group, which followed a slightly skewed (skewness = –1.04) and non-normal distribution (Shapiro–Wilk test: *p* = 0.03), we did not apply any data-transformation before hypothesis testing.

We tested the first hypothesis that variable training leads to larger structure-independent, inter-manual, and sequence-specific transfer using three repeated-measures general linear model (GLM) analyses. In each model we regressed the mean RT for the corresponding sequence on the within-subject factor Session (Baseline, Transfer), the between-subjects factor Group (Constant, Variable), and the Session × Group interaction term. We further tested whether the amount of transfer was related to improvements in the training sequence by correlating – in each training group – the Improvement Scores of the transfer sequences (T0, TrL, and T3) with the Improvement Scores of Tr1.

To test the second hypothesis that sequence-specific knowledge transfers to a novel but structurally overlapping sequence we first compared the amount of RT improvement in the triplet-sharing sequence (T3) with RT improvements in the non-overlapping sequence (T0). We used a repeated-measures GLM for Improvement Scores with Transfer Sequence (T0 or T3) as within-subject factor, Group (Constant or Variable) as between-subject factor, and the Transfer Sequence × Group interaction term. To investigate whether sequence knowledge was expressed specifically for overlapping triplets we directly compared Improvement Scores for familiar and unfamiliar sequence transitions. Given that all sequences were second-order conditional sequences the identity of the third triplet element of shared triplets is predictable because it is always preceded by the same two elements, independent of the triplet’s ordinal position within the sequence. Comparing RT improvements for such predictable elements with RT improvements for the same, but non-predictable elements (i.e., same key presses but within an unfamiliar triplet) provides a specific estimate of sequence transfer. **Table 1** shows the familiar triplets (with predictable third elements) in T3 and the corresponding unfamiliar triplets (with non-predictable elements) in T3 and T0. For example, element “4” in T3 is predictable when it is preceded by “1-3” (because the triplet “1-3-4” is shared with Tr1), but not when it is preceded by “3-2” or by “4-1”. We thus calculated three separate averages of Improvement Scores for each participant, one for predictable elements in T3, one for corresponding non-predictable elements in T3, and one for the corresponding non-predictable elements in T0. These averages were entered into a repeated-measures GLM with within-subject factor Element type (T3-Predictable, T3-Non-predictable, T0-Non-predictable), between-subjects factor Group (Constant or Variable), and the Transition type × Group interaction. Subsequent pairwise comparisons between the different levels of Transition type were corrected for multiple comparisons using Bonferroni correction.

**Table 1:** Familiar and corresponding unfamiliar triplets.

Familiar triplets (in Tr1 and T3)	Unfamiliar triplets (in T3 and T0)
1-3-4	3-2-4, 4-1-4
4-3-2	3-1-2, 1-4-2
2-3-1	1-2-1, 2-4-1

*Familiar triplets were shared between Tr1 and T3, unfamiliar triplets were shared between T3 and T0. The last elements of familiar triplets were predictable and each of them had two corresponding unpredictable elements in the unfamiliar triplets of T3 and T0. Three different averages were computed from the RTs of the last (underlined) elements of these triplets: (1) average RTs of predictable elements in T3, (2) average RTs of corresponding unpredictable elements in T3, and (3) average RTs of corresponding unpredictable elements in T0.*

For hypotheses where we predicted an effect in a particular direction we used one-tailed levels of significance at α = 0.05 to maximize power. Where applied, the usage of one-tailed tests is stated in the results. All other tests were performed using two-tailed levels of significance at α = 0.05.

## Results

### Performance Improvements on Trained Sequences

**FIGURE 3:**
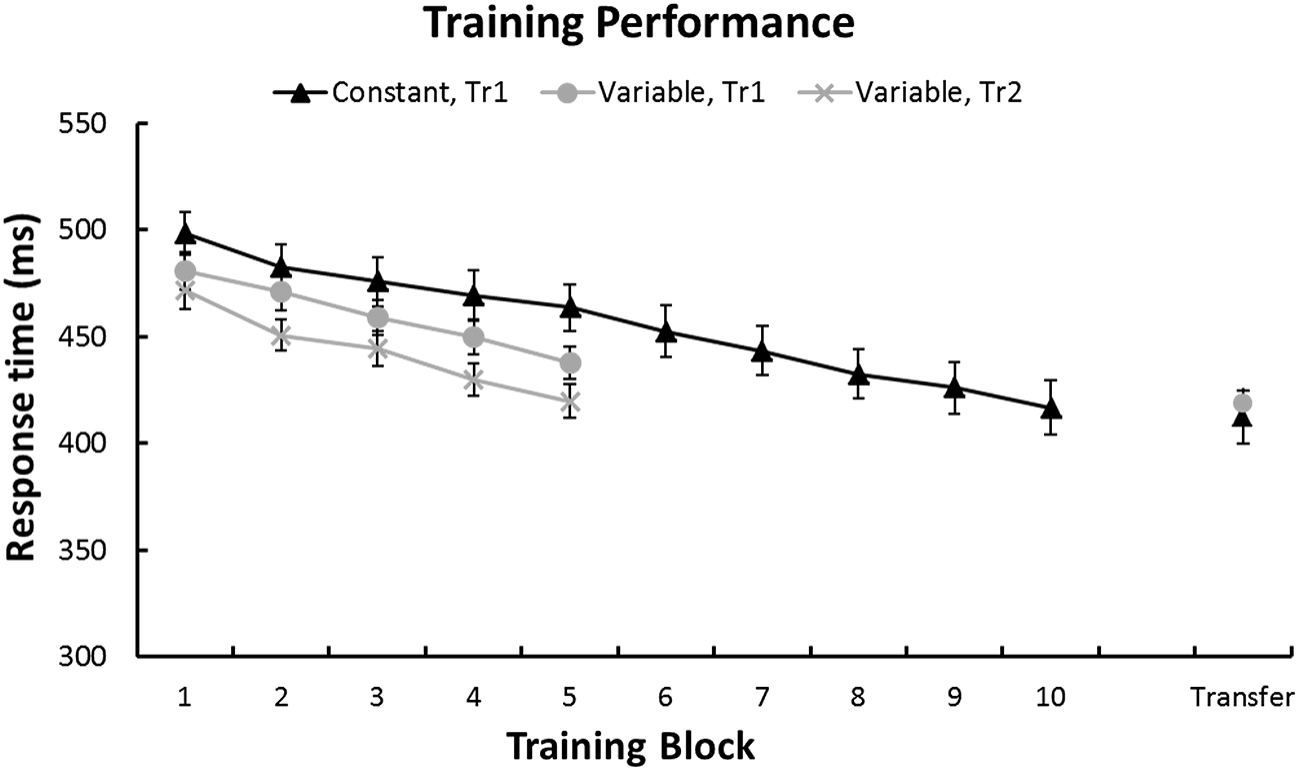
**Performance during training.** In both groups RTs decreased linearly during training. Error bars represent standard error of the mean. Note that in the Variable group, blocks of Tr1 and Tr2 training were interleaved.

**FIGURE 4:**
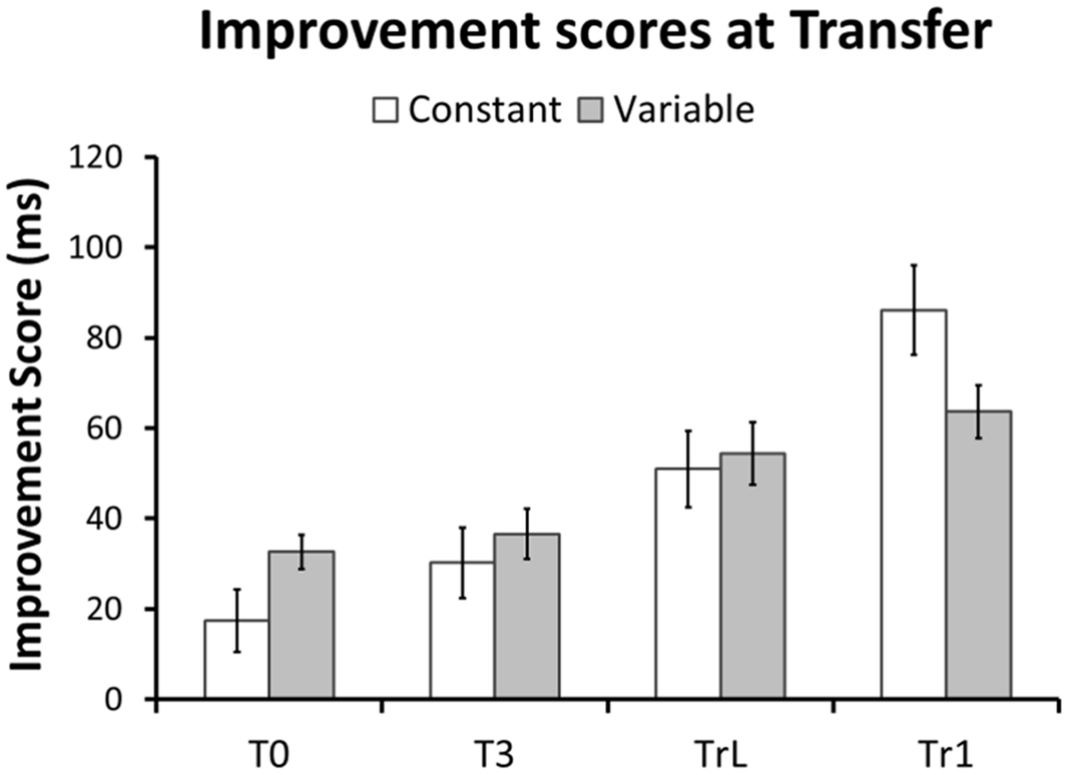
**Performance improvements at Transfer.** Improvements in RT are shown as between-participant means of the within-participant difference between median RT at Baseline and Transfer. Note that in both groups and for all sequences RTs improved after training. Error bars represent standard error of the mean.

First, to confirm that sequence-learning was successful we performed a repeated-measures GLM on the RTs for each training sequence and Group. **Figure 3** shows a continuous RT decrease across training blocks for each sequence. This was confirmed by linear within-subjects contrasts: Tr1, Constant group [*F*(1,27) = 62.6, *p* < 0.001], Tr1, Variable group [*F*(1,27) = 93.1, *p* < 0.001], and Tr2, Variable group [*F*(1,27) = 67.1, *p* < 0.001]. Furthermore, a repeated-measures GLM with factors Session (Baseline/Transfer) and Group (Constant/Variable) confirmed that in both groups RTs for Tr1 were significantly reduced at Transfer [main effect of Session: *F*(1,54) = 127.3, *p* < 0.001]. Additionally, there was an interaction between Group and Session [*F*(1,54) = 10.4, *p* = 0.002], with greater post-training RT improvements in the Constant training group. Improvement Scores (i.e., Baseline RTs – Transfer RTs) for Tr1 and for the three transfer sequences, T0, T3, and TrL are shown in **Figure 4** and mean RTs per Group and Session (Baseline and Transfer) are shown in **Table 2**.

**Table 2:** Response times per group during Baseline and Transfer sessions.

	Constant		Variable	
Task	Baseline	Transfer	Baseline	Transfer
Tr1	498 ± 53	412 ± 66	482 ± 46	419 ± 38
TrL	534 ± 65	483 ± 59	519 ± 50	464 ± 43
T0	507 ± 60	490 ± 57	494 ± 35	461 ± 37
T3	493 ± 57	463 ± 59	483 ± 45	446 ± 39

*Response times (in ms ± SD) show the group means of the median RTs of each participant. In both groups and for all sequences, RTs decreased significantly from Baseline (i.e., first block) to Transfer (i.e., last block) sessions (all *p* < 0.01, except for T0 in the Constant group, where *p* = 0.019).*

### Variable Training and Transfer

**FIGURE 5:**
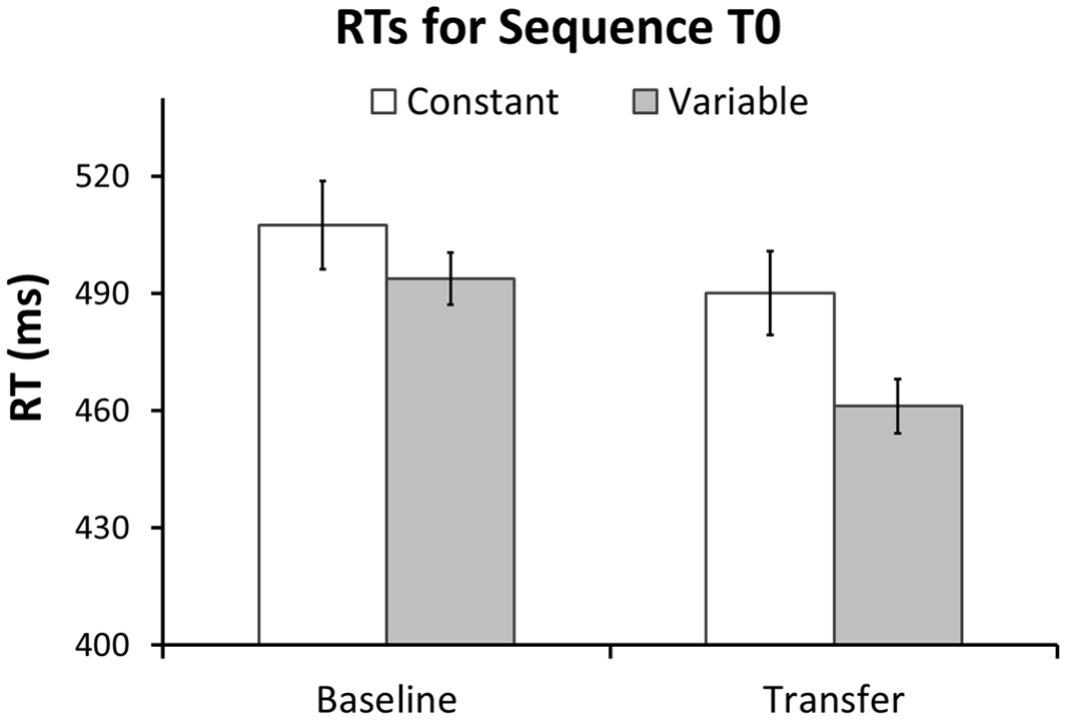
**Transfer effects on sequence T0 after constant and variable training.** Mean response times at Baseline and Transfer for sequence T0 are shown separately for the each group. The Variable group showed a significantly larger RT reduction at Transfer than the Constant group. There were no significant group differences in RT at Baseline. Error bars represent standard error of the mean.

**FIGURE 6:**
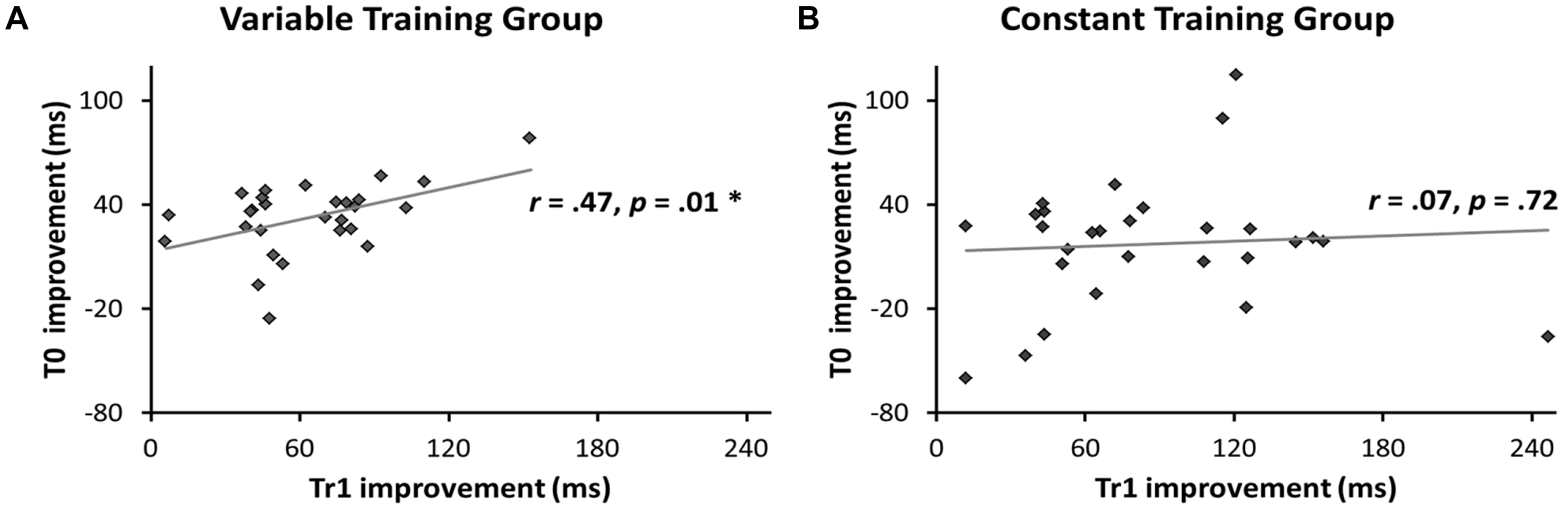
**Correlations between improvement scores on the training sequence Tr1 and the transfer sequence T0.** Improvement scores were based on RT differences between the first block and the Transfer block of each sequence. Correlations between improvement scores for Tr1 and T0 were calculated separately for the Variable **(A)** and the Constant **(B)** group. Only the Variable group showed a significant correlation.

Our first hypothesis was that the different types of transfer (i.e., sequence non-specific, inter-manual, and sequence-specific transfer) are larger after variable than after constant training. First, we predicted that the Variable group would have larger transfer to T0 than the Constant group. In line with this prediction the GLM for RTs in T0 revealed a greater RT reduction between sessions in the Variable than in the Constant group, as evident by a significant Session × Group interaction in the predicted direction [*F*(1,54) = 3.7, *p* = 0.03, one-tailed]. Further, there was a significant RT improvement across sessions [main effect of Session: *F*(1,54) = 39.7, *p* < 0.001], but no significant difference between groups [main effect of Group: *F*(1,54) = 2.98, *p* = 0.09]. **Figure 5** shows mean RTs per Group and Session for the T0 sequence. To test whether these differences in RT improvement could be influenced by differences in accuracy we performed the same GLM with factors Session, Group and the Group × Session interaction on the number of errors in T0. The amount of errors did not differ between groups [main effect of Group: *F*(1,54) = 0.73, *p* = 0.40], nor was there an interaction between Group and Session [*F*(1,54) = 0.60, *p* = 0.44]. However, a significant main effect of Session [*F*(1,54) = 31.6, *p* < 0.001] revealed that both groups committed more errors at Transfer than at Baseline (see **Figure 2**). Further, we tested whether, on the between-participant level, performance improvements in the transfer sequence T0 were related to the magnitude of improvement in the trained sequence Tr1. Improvement scores for T0 and Tr1 were significantly correlated in the Variable group (*r* = 0.47, *p* = 0.01; **Figure 6A**) but not in the Constant group (*r* = 0.07, *p* = 0.72; **Figure 6B**). One participant in the Constant group had a somewhat extreme RT improvement in Tr1 of 246.5 ms and might be considered an outlier (see **Figure 6B**). To make sure that the correlation in the Constant group was not distorted by this single value we repeated the same analysis under exclusion of this data point. Removing this value did not change the result, as the correlation remained non-significant (*r* = 0.32, *p* = 0.11). Further, since the Constant group received five more blocks of Tr1 training than the Variable group, one might argue that for the Constant group, a correlation with T0 improvements might rather be present in the first five blocks of Tr1 training. However, no correlation with T0 improvements was found also when using the Improvement Scores of only the first five Tr1 blocks (*r* = 0.01, *p* = 0.96).

Effects of variable training on inter-manual transfer were tested analogously. Contrary to our expectation there was no significant Session × Group interaction [*F*(1,52) = 0.10, *p* = 0.38, one-tailed]. RTs decreased significantly between sessions [main effect of Session: *F*(1,52) = 94.31, *p* < 0.001] and there was no significant difference between groups [*F*(1,52) = 1.55, *p* = 0.22]. The number of errors did not differ between Groups [*F*(1,52) = 0.12, *p* = 0.73] or Sessions [*F*(1,52) = 0.42, *p* = 0.52], nor was there an interaction [*F*(1,52) = 1.13, *p* = 0.30]. Improvement Scores for TrL were not correlated with improvements inTr1, neither in the Variable (*r* = 0.31, *p* = 0.11), nor in the Constant (*r* = 0.15, *p* = 0.46) group.

Finally, we used the same approach to test effects of variable training on sequence-specific transfer in T3. Contrary to our expectation there was no significant Session × Group interaction [*F*(1,52) = 0.45, *p* = 0.25, one-tailed]. Again, RTs decreased significantly across sessions [main effect of Session: *F*(1,54) = 48.56, *p* < 0.001] and there was no significant difference between groups [*F*(1,52) = 1.14, *p* = 0.29]. Similar to the other transfer sequences the number of errors did not differ between groups [*F*(1,54) = 2.45, *p* = 0.12], nor did it show a Group × Session interaction [*F*(1,54) = 2.44, *p* = 0.12]. However, participants made more errors at the transfer than at baseline session [main effect of session: *F*(1,54) = 44.48, *p* < 0.001] (see **Figure 2**). Finally, we tested whether Improvement Scores for T3 were correlated with improvements in Tr1 at the between-participant level. The Variable group showed a strong trend toward a positive correlation (*r* = 0.37, *p* = 0.055), but there was no correlation in the Constant group (*r* = –0.23, *p* = 0.24).

### Sequence-specific Transfer

The second hypothesis was that sequence-specific knowledge acquired during training can be used in the context of a novel sequence. To test this, we first investigated whether transfer effects were larger for the sequence that shared triplets with the trained sequence (T3) than for the sequence that had no structural overlap with the trained sequence (T0). We examined this using a repeated-measures GLM for Improvement Scores with the factors Transfer Sequence (T0/T3), Group (Constant/Variable), and the Transfer Sequence × Group interaction term. In line with the hypothesis, we found an effect of Transfer Sequence in the predicted direction (i.e., larger improvement for T3 than for T0) [*F*(1,54) = 4.06, *p* = 0.025, one-tailed]. There was no effect of Group [*F*(1,54) = 1.95, *p* = 0.17] nor a Transfer Sequence × Group interaction [*F*(1,54) = 1.12, *p* = 0.30].

**FIGURE 7:**
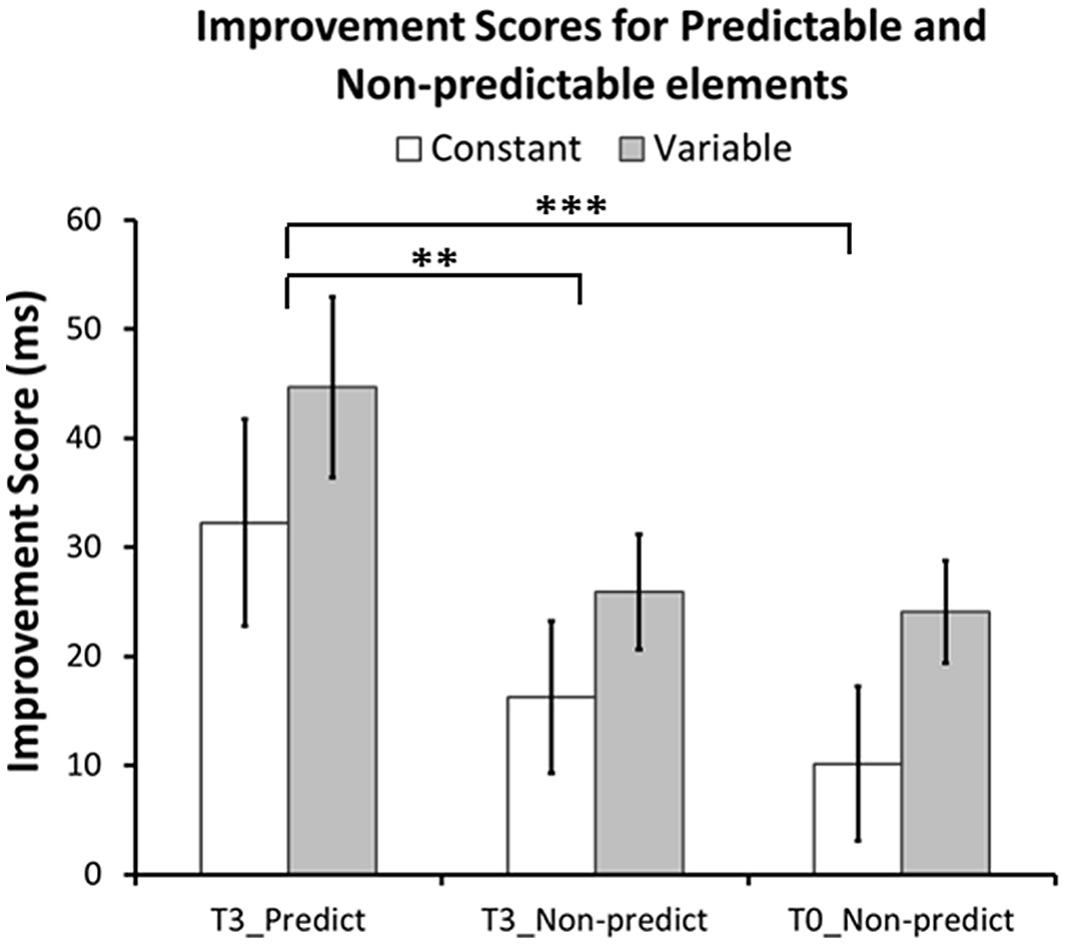
**Performance improvements for predictable and non-predictable elements.** Improvement scores for predictable elements are derived from the average improvement scores from the last elements of familiar triplets in T3. Improvement scores for non-predictable elements are derived from the average improvement scores from corresponding elements (i.e., same finger) of unfamiliar triplets in T3 and T0. In both groups, performance increased more for predictable elements (in T3) than for corresponding non-predictable elements (in both T3 and T0). There was no difference between non-predictable elements in T3 and T0. None of the comparisons revealed a significant group or interaction effect. ^∗∗^*p* = 0.004, ^∗∗∗^*p* = 0.001, Bonferroni corrected

As a more precise test for sequence-specific transfer, we investigated whether predictable sequence elements (last element of familiar triplets) showed greater RT improvements after training than corresponding non-predictable elements (last element of unfamiliar triplets). A GLM analysis of the Improvement Scores with within-subject factor Element Type (T3-Predictable, T3-Non-predictable, T0-Non-predictable), between-subjects factor Group (Constant, Variable), and the Element × Group interaction revealed a significant main effect of Element Type [*F*(2,107) = 10.0, *p* < 0.001, one-tailed], but no effect of Group [*F*(1,54) = 2.1, *p* = 0.15], or of the Element Type × Group interaction [*F*(2,107) = 0.09, *p* = 0.91]. Bonferroni-corrected pairwise comparisons further confirmed that predictable elements in T3 showed greater RT improvements than corresponding non-predictable elements in both T3 [*F*(1,54) = 11.8, *p* = 0.004] and T0 [*F*(1,54) = 16.2, *p* = 0.001]. RT improvements for non-predictable elements in T3 and T0 did not differ from each other [*F*(1,54) = 0.68, *p* = 1.0]. There were no differences between or interactions with Group in any of the comparisons (T3-Predictable vs. T3-Non-predictable Group effect: [*F*(1,54) = 1.32, *p* = 0.78], interaction: [*F*(1,54) = 0.08, *p* = 1.0]; T3-Predictable vs. T0-Non-predictable Group effect: [*F*(1,54) = 2.0, *p* = 0.48], interaction: [*F*(1,54) = 0.02, *p* = 1.0]; T3-Non-predictable vs. T0-Non-predictable Group effect: [*F*(1,54) = 2.7, *p* = 0.33], interaction: [*F*(1,54) = 0.20, *p* = 1.0]). **Figure 7** shows that for both groups Improvement Scores for predictable elements are larger than those for non-predictable elements.

### Sequence Awareness

The two groups did not differ on either of the two sequence awareness measures. In both the Constant and the Variable group, 19 out of 28 participants answered “Yes” to the first question on whether they had noticed any pattern in the stimulus presentation. Further, there was no group difference in participants’ response to the second question on how sure (1 = “not sure at all”; 10 = “very sure”) they were of the presence of a sequential pattern [Constant: mean = 6.89, *SD* = 2.81; Variable: mean = 6.75, *SD* = 3.04; *t*(54) = 0.18, *p* = 0.86].

## Discussion

We investigated two hypotheses about transfer of motor sequence skills. First, we tested whether variable practice leads to greater skill transfer than constant practice by examining the effect of practice schedule on three different types of transfer: task-general, inter-manual, and sequence-specific transfer. Second, we tested if structure-specific sequence knowledge can transfer to a novel sequence context. In partial support of our first hypothesis, we found greater transfer after variable than after constant practice, but only for the structurally non-overlapping (T0) sequence and not for the structurally identical inter-manual (TrL) and the triplet-sharing (T3) transfer sequences. Variable practice was thus advantageous for task-general transfer but not for inter-manual or sequence-specific transfer. Our second hypothesis that fragments of sequence-specific knowledge can transfer to a novel sequential context was supported by two observations. First, transfer was larger for the triplet-sharing (T3) sequence than for the non-overlapping (T0) sequence. Moreover, within the triplet-sharing sequence transfer was larger for elements that were predictable based on previously acquired sequence knowledge (i.e., the third element of shared triplets) than for elements that were not predictable (i.e., corresponding elements of non-shared triplets).

### Practice Schedule and Sequence-unspecific Transfer

The presence of transfer effects to the structurally non-overlapping sequence T0 indicates that task-general skills (i.e., skills that are independent of sequence structure) contributed to skill transfer. Performance of any SRT paradigm requires stimulus perception, response selection and generation, and the formation of correct stimulus–response associations. Training could improve one or several of these basic task components. This likely explains why even RTs on random SRT sequences improve with practice (Thomas and Nelson, 2001; Robertson, 2007; Song et al., 2015). But why is transfer of task-general skills larger after variable than after constant training, despite of equal amounts of practice with the SRT task?

Generally, this finding is in agreement with transfer benefits of variable practice that have been reported for various real-life skills such as, e.g., tennis (Douvis, 2005), wheelchair driving (Yao et al., 2009), and rotatory pursuit skills (Heitman et al., 2005). However, in the present task both practice schedules provided exactly the same amount and format of task-general practice, making it unlikely that one group would have truly improved more on non-specific skills such as stimulus processing or stimulus–response mapping. This suggests that, somewhat paradoxically, differences in task-general transfer might have resulted from differences in the exposure or learning of sequential structures.

One possible explanation for this could be that the transfer differences between training groups reflect differences in their susceptibility to interference from negative transfer. Negative transfer has been found, e.g., in sequential rule application paradigms where a set of number manipulation rules has to be applied in a specific order to solve a cognitive task (Woltz et al., 2000). The more training participants received in that task, the more errors they made on a transfer task in which the same rules had to be applied in a different order. Participants were often unaware of their errors, suggesting that the errors reflect the involuntary behavioral expression of implicit sequence representations, which are inaccessible to conscious control. A similar phenomenon has been described for SRT paradigms where RTs of random order trials are slowed down by the previous execution of sequential trials (Robertson, 2007). Again, this suggests that also for motor sequences expectations about sequence order can interfere with performance at transfer. In line with these findings, we observed that the Constant practice group showed less skill transfer to the non-overlapping sequence, despite of larger performance improvements during training. Thus, greater sequence knowledge may have caused more interference with a non-overlapping sequence at transfer. The Variable group had the same total amount of SRT practice, but the alternating training schedule may have led to weaker or more flexible sequence expectations. Thus, while both variable and constant practice promote transfer of task-general skills, constant practice may limit the total amount of transfer due to interference from violated sequence expectations. Such differences in the susceptibility to negative transfer would also explain why the amount of transfer correlated with training improvements only in the Variable and not in the Constant practice group. If constant practice increases both task-general skills and sequence-specific expectations, then improvements during training will simultaneously increase positive and negative transfer thereby precluding a direct relation between training improvements and transfer.

The finding that after training, both groups made more errors on the T0 and T3 sequences suggests that negative transfer also affects performance accuracy. This post-training decrease in T0 and T3 accuracy cannot be explained by a general performance drop toward the end of the experiment because accuracy on the perceptually familiar sequences (both Tr1 and TrL) did not decrease at transfer. Further, it is important to note that the interference with accuracy was similar in both groups, making it unlikely that the observed differences in RT transfer were due to differences in accuracy.

### Practice Schedule and Inter-manual Transfer

Both groups showed inter-manual transfer, as evident in performance improvements with the untrained (left) hand after training. Hikosaka et al. (2002) suggested that sequence learning may involve the acquisition of multiple sequence representations: a rapidly acquired, effector-independent representation in extrinsic visuo-spatial coordinates and a more gradually acquired effector-specific representation in intrinsic motor coordinates (Hikosaka et al., 2002). According to this model, the relatively short training period in the current task would have promoted predominantly effector-independent representations in extrinsic coordinates (see also: Shea et al., 2011). Inter-manual transfer would thus require the remapping of external response locations to a new set of motor commands for the opposite hand. Similar to the present results, earlier experiments also found large inter-manual transfer effects for sequences of the same perceptual structure (Japikse et al., 2003; Kovacs et al., 2009), suggesting that inter-manual transfer makes use of sequence representations in extrinsic space.

However, contrary to our hypothesis, we observed no difference in inter-manual transfer between the Constant and the Variable group. This suggests that practice schedule has a weak or no influence on inter-manual transfer and that constant and variable practice do not cause different amounts of interference for the untrained hand. In fact, given that the inter-manual transfer sequence was perceptually identical to the training sequence, any involuntary expression of perceptual sequence knowledge would have contributed positively to transfer performance. It would be interesting to investigate in future studies whether negative inter-manual transfer can be seen for sequences that are perceptually different but motorically identical (i.e., require the same sequence of finger movements) or homologous (i.e., require the same finger movements but with the opposite hand). A recent study by Handa et al. (2015) showed that overnight oﬄine gains after sequence practice are reduced when either an entirely novel or a motorically similar (but perceptually different) sequence is practiced immediately after the target sequence. Interestingly, oﬄine gains were not impaired after adding a visuo-spatially identical sequence at the end of training (Handa et al., 2015). These findings are in agreement with the present results in that they suggest that interference – both with immediate transfer and with consolidation – is larger for the perceptual than for the motoric component of motor sequencing tasks.

### Practice Schedule and Sequence-specific Transfer

Both practice groups showed transfer to the triplet-sharing sequence T3, but contrary to our expectation the amount of transfer did not differ between groups. Based on a recent study by Song et al. (2015), which showed that memories for movement transitions were improved after variable compared with constant practice, we expected performance on the triplet-sharing sequence to show similar advantages of variable practice. However, there are several differences between the two studies that could explain this discrepancy. First, Song et al. (2015) evaluated sequence performance after 30 min and 1 week retention periods, whereas transfer in the present study was evaluated immediately after sequence training. It may be possible that sequence-specific benefits of variable practice need an, albeit short (30 min), consolidation period to take effect. Another difference is that Song et al. (2015) used random sequences, both for alternation with the training sequence in the Variable condition and for evaluation of transfer performance. In their study, transfer was quantified by comparing performance on triplets in the random transfer sequence that also appeared in the training sequence (i.e., ‘familiar’ triplets) with triplets in the random sequence that did not appear in the training sequence (i.e., ‘unfamiliar’ triplets). In the present study both the alternating training sequence and the transfer sequence were repeating sequences that were specifically designed to match the training sequence in terms of salient statistical properties and overlapping triplets. It is possible that interleaving sequence practice with random sequences has different effects on sequence learning and transfer than interleaving sequence practice with another sequence. It would be interesting to directly compare these two methods of creating alternated training schedules (i.e., alternation with a random sequence vs. alternation with another deterministic sequence).

Finally, one might argue that evaluating transfer based on only the overlapping sub-sequences (as in Song et al., 2015) rather than on the entire transfer sequence would yield a more accurate quantification of sequence-specific knowledge. However, also the specific comparison of improvement scores on familiar and unfamiliar element transitions did not reveal any differences between the Constant and the Variable practice group (see further discussion below).

### Transfer of Sequence-specific Knowledge

In support of our second hypothesis, we found sequence-specific transfer effects in addition to sequence-unspecific transfer. Sequence-specific transfer was demonstrated by two observations. First, transfer sequence T3, which shared sequence structure with the training sequences (Tr1 and Tr2), showed larger transfer effects than the sequence without structural overlap (T0). Importantly, transfer sequences T0 and T3 were constructed to have identical lower-level statistical properties, with the key difference between them being that T3, but not T0, contained trained subsequences (i.e., triplets). This strongly suggests that the performance advantage for T3 at transfer was mediated by familiar sequence fragments contained in T3, but not in T0.

This interpretation is further supported by the results of the element-specific analysis. Because both training sequences (Tr1 and Tr2) were constructed to share the same three triplets with transfer sequence T3, we were able compare RT improvements of predictable elements with those of corresponding non-predictable elements. As expected, transfer was larger for predictable elements in T3 than for corresponding non-predictable elements in T3 and T0. A control analysis showed that this was not due to non-specific RT differences between T3 and T0 because improvements for non-predictable elements were similar in both sequences. We did not observe any difference between practice groups or interaction between groups and element-specific improvements. This was in line with our previous results where practice schedule did not seem to affect transfer to the entire T3 sequence. In contrast to the study by Song et al. (2015), we considered only the last element and not the entire shared triplet as familiar. This was because in a second order conditional sequence each element is only determined by its two preceding elements. Thus, if a familiar triplet is placed into a novel sequence context the first two triplet elements are necessary (and sufficient) to predict the third element, but the first two elements themselves are not predictable. Further, it is important to note that the non-predictable elements that were used for comparison with predictable elements required the same button presses (i.e., same finger movements). This excludes the possibility that the observed effect was confounded by simple RT differences between fingers (Lachnit and Pieper, 1990).

The sequence-specific transfer effects resemble *part-whole* transfer where serial task performance is facilitated by previous training of the elemental tasks of a sequence (Schmidt and Lee, 2005; Spruit et al., 2014). Such part-task practice has been commonly studied in relation to complex and difficult real life skills, such as industrial tasks (Seymour, 1954; So et al., 2013), surgery (Dankelman et al., 2005; Spruit et al., 2014), and aircraft control (Adams and Hufford, 1962) and is also an important practice strategy in stage arts like music and dance. In motor-sequence learning paradigms part-whole transfer has been demonstrated for spatiotemporal sequences, where knowledge about the ordinal structure of a sequence partially transferred to sequences with the same ordinal, but a different temporal structure (Ullén and Bengtsson, 2003; Sanchez et al., 2015). Finally, the present results extend the previously described findings by Jiménez et al. (2006), in demonstrating sequence-specific skill transfer after incidental learning even when short sequence fragments (triplets) are taken out of their familiar sequence context and embedded within a novel sequence. Although we did not precisely quantify the amounts of explicit and implicit sequence knowledge it seems likely that triplet-specific sequence transfer was largely implicit since it would be very difficult to identify three overlapping triplets at shifted ordinal positions within a single block of a looping transfer sequence (see Limitations for a further discussion of explicit vs. implicit knowledge).

### Limitations

One limitation of the present study is that even though an incidental learning paradigm was employed, we cannot distinguish between transfer of explicit and implicit sequence knowledge. Given that the SRT task is considered to be not a purely implicit learning task (Willingham and Goedert-Eschmann, 1999; Moisello et al., 2009; Abrahamse et al., 2010) and that complete absence of explicit knowledge is difficult to demonstrate (Wilkinson and Shanks, 2004; Abrahamse et al., 2010), it was beyond the scope of the present study to differentiate between the contributions of implicit and explicit sequence knowledge. However, more than two thirds of our participants indicated to have noticed some regularity in the stimulus presentation, suggesting that skill transfer may have involved some explicit knowledge. To differentiate between practice schedule effects on more implicit or explicit knowledge transfer it would be necessary to conduct further studies that directly manipulate the amount of explicit sequence knowledge between participants.

One possible confound in the present study might have been if participants in the Constant practice group developed greater explicit sequence knowledge than those in the Variable group. In this case, group differences in skill transfer could have reflected differences in sequence awareness. However, we think that this is unlikely for two reasons: first, the two groups did not differ in their answers to the sequence awareness questions and second, previous studies have generally not found any relations between explicit sequence knowledge and the amount of skill transfer (Song et al., 2012; Sanchez et al., 2015).

For inter-manual transfer the present design does not allow us to distinguish between contributions from sequence-specific and task-general transfer effects. A non-overlapping transfer sequence for the left hand would have been necessary to control for sequence non-specific inter-manual transfer. However, given the strict constraints on the statistical regularities of our second order conditional sequences we were unable to construct a suitable non-overlapping sequence for the left hand. For future studies it would thus be interesting to include such an inter-manual control sequence to test whether practice schedule affects the task-general component of inter-manual transfer in a similar way as it affected task-general transfer in the trained hand.

Another limitation of this study is that transfer performance was evaluated immediately after training, but not at an additional later time point. In a comprehensive review on the distinction between measures of motor skill learning and performance Kantak and Winstein (2012) point out that delayed retention (e.g., performance measured after 24 h) is a better indicator for motor learning than performance measured immediately at the end of training. The authors argue that performance during acquisition/ at the end of training is influenced by various transient factors that are not reflective of the more permanent performance changes that are indicative of motor learning. Furthermore, different training schedules may have different effects on the mechanisms and neural substrates of skill consolidation (see Kantak et al., 2010; Tanaka et al., 2010). Such effects on the consolidation, rather than the encoding stage can only be detected if performance is assessed after a time delay (e.g., 4–6 h) that allows for consolidation to take place (Kantak et al., 2010). Although the aim of the current study was to evaluate transfer of motor skills, rather than motor learning *per se*, our measures of transfer may have been similarly affected by the presence of transient factors or by the absence of a consolidation period. In fact, the decrease in task-general transfer after constant practice was likely due to such a transient factor at the time of practice (i.e., interference due to acquired sequence expectations). An additional transfer evaluation after a delay period would have provided more information about the temporal persistence of these interference effects. Thus, it would be interesting to investigate in future studies if the effect of practice schedule on sequence-specific transfer differs before and after a consolidation period.

## Conclusion

Using specifically constructed sequences we were able to show differential effects of practice schedule on different types of skill transfer. A constant practice schedule reduced task-general, but not inter-manual or sequence-specific transfer, suggesting that negative transfer may be an important factor to take into account when similar transfer tasks are performed immediately after a blocked training session. Further, we found that structure-specific knowledge can transfer between sequences, even if the transfer sequence contains only short (i.e., three elements-long) sub-sequences that are embedded within a new sequential context. This finding has an important implication for the design of future SRT paradigms, because it suggests that performance comparisons between training and test sequences should take into account that sequence-specific knowledge may transfer even to short segments of structural overlap that are commonly present in random control sequences.

## Author Contributions

DM and FU designed the study. DM acquired, analyzed, and interpreted the data. The manuscript was written by DM and revised by FU. Both authors approved the final version of the manuscript.

## Conflict of Interest Statement

The authors declare that the research was conducted in the absence of any commercial or financial relationships that could be construed as a potential conflict of interest.
